# Incretin-Based Therapies in Obesity-Related Heart Failure With Preserved Ejection Fraction (HFpEF): A Systematic Review of Emerging Cardiometabolic Disease Modification Beyond Glycemic Control

**DOI:** 10.7759/cureus.109953

**Published:** 2026-05-31

**Authors:** Vaishnavi Hemdev, Mohammad Abuzenah, Qossay Alsaafin, Zaid Al Ghananeem, Fnu Diksha, Adnan Khatak

**Affiliations:** 1 Internal Medicine, University of York, York, GBR; 2 Internal Medicine, Salford Royal NHS Foundation Trust, Salford, GBR; 3 Acute Medicine, Royal Albert Edward Infirmary, Wigan, GBR; 4 Internal Medicine, Aneurin Bevan University Health Board, Newport, GBR; 5 Internal Medicine, People's University of Medical and Health Sciences for Women, Nawabshah, PAK; 6 Internal Medicine, Allama Iqbal Medical College, Lahore, PAK

**Keywords:** cardiometabolic heart failure, glp-1 receptor agonists, incretin-based therapy, obesity, semaglutide, tirzepatide

## Abstract

Heart failure with preserved ejection fraction (HFpEF) remains a clinically heterogeneous syndrome with limited disease-modifying therapeutic options, particularly among patients with obesity-related cardiometabolic dysfunction. Increasing evidence suggests that obesity-associated HFpEF represents a distinct inflammatory and metabolically active phenotype characterized by visceral adiposity, endothelial dysfunction, congestion physiology, impaired exercise capacity, and adverse cardiac remodeling. Incretin-based therapies, including glucagon-like peptide-1 receptor agonists and dual glucose-dependent insulinotropic polypeptide/glucagon-like peptide-1 receptor agonists, have recently emerged as promising interventions within this evolving therapeutic landscape. This systematic review evaluated contemporary randomized clinical evidence examining the effects of semaglutide and tirzepatide in obesity-related HFpEF. A comprehensive literature search was conducted across PubMed/MEDLINE, Scopus, and Web of Science for studies published between January 2020 and July 2025. Nine studies met the predefined eligibility criteria, including landmark randomized controlled trials, pooled analyses, and mechanistic imaging and biomarker substudies. Across the included studies, incretin-based therapies consistently improved heart failure-related symptoms, exercise capacity, quality of life, inflammatory biomarkers, and body weight, while also demonstrating favorable effects on structural remodeling, congestion-related physiology, and cardiovascular-kidney interactions. Mechanistic analyses suggested potential benefits involving reductions in left ventricular mass, paracardiac adipose tissue, inflammatory burden, plasma volume expansion, and markers of myocardial and renal injury. Collectively, the current evidence supports the growing role of incretin-based therapies as promising phenotype-oriented interventions in obesity-related HFpEF and raises the possibility that targeted cardiometabolic modulation may influence multiple domains of disease pathophysiology beyond glycemic control alone. However, further long-term studies are needed to clarify their effects on remodeling reversal, arrhythmia burden, and cardiovascular mortality.

## Introduction and background

Heart failure with preserved ejection fraction (HFpEF) has emerged as one of the most complex and rapidly expanding cardiovascular syndromes worldwide, accounting for nearly half of all heart failure cases and contributing substantially to morbidity, hospitalization burden, and impaired quality of life [1,2]. Unlike heart failure with reduced ejection fraction, HFpEF encompasses a heterogeneous spectrum of pathophysiologic phenotypes characterized by varying degrees of metabolic dysfunction, systemic inflammation, endothelial impairment, myocardial remodeling, and multiorgan involvement. This heterogeneity has contributed to persistent therapeutic challenges, as many conventional heart failure strategies developed within predominantly ischemic or reduced ejection fraction paradigms have demonstrated limited and inconsistent efficacy across broader HFpEF populations [3]. In particular, traditional hemodynamic therapies may inadequately address the inflammatory, metabolic, and adiposity-driven mechanisms that appear central to obesity-related HFpEF pathophysiology.

Among the recognized HFpEF phenotypes, obesity-related HFpEF has gained increasing attention because of its distinct clinical and biologic profile. Obesity contributes not only to increased hemodynamic load and plasma volume expansion but also to chronic low-grade inflammation, visceral adiposity, impaired skeletal muscle energetics, endothelial dysfunction, and adverse myocardial remodeling [4]. Patients with obesity-related HFpEF frequently demonstrate marked exercise intolerance, elevated filling pressures, systemic inflammatory activation, and a high prevalence of associated cardiometabolic conditions, including type 2 diabetes mellitus (T2DM) and chronic kidney disease (CKD). These observations have led to growing recognition that obesity-related HFpEF may represent a metabolically active and inflammation-driven subtype of heart failure requiring more phenotype-oriented therapeutic approaches [5].

Incretin-based therapies, particularly glucagon-like peptide-1 (GLP-1) receptor agonists and dual glucose-dependent insulinotropic polypeptide/glucagon-like peptide-1 (GIP/GLP-1) receptor agonists, have recently emerged as promising interventions within this evolving therapeutic landscape [6]. Initially developed for glycemic management and obesity treatment, these agents have demonstrated broader cardiometabolic effects involving weight reduction, inflammatory modulation, improved exercise capacity, and favorable changes in cardiovascular and renal physiology [7]. Although both therapeutic classes share overlapping metabolic and anti-inflammatory effects, pure GLP-1 receptor agonists primarily exert their benefits through appetite suppression, weight reduction, endothelial improvement, and modulation of inflammatory signaling, whereas dual GIP/GLP-1 agonists may additionally enhance adipose tissue regulation and insulin sensitivity through complementary GIP-mediated mechanisms, potentially amplifying effects on congestion biology, hemodynamics, and adverse cardiac remodeling. Recent randomized trials evaluating semaglutide and tirzepatide in obesity-related HFpEF have reported improvements in symptoms, physical function, quality of life, congestion-related parameters, and markers of structural remodeling. Importantly, several mechanistic analyses have suggested that these effects may extend beyond glycemic control alone, raising important questions regarding the potential disease-modifying role of incretin-based therapies within obesity-related HFpEF phenotypes [8]. In this context, disease modification refers not only to secondary hemodynamic improvement from weight reduction but also to the potential alteration of inflammatory, structural, metabolic, and myocardial remodeling pathways underlying obesity-related HFpEF.

Given the rapidly evolving evidence base and the growing interest in phenotype-specific management strategies, this systematic review aims to evaluate the current clinical and mechanistic evidence surrounding incretin-based therapies in obesity-related HFpEF. The review specifically focuses on the multidomain effects of semaglutide and tirzepatide on symptoms, exercise function, inflammatory burden, structural remodeling, congestion physiology, and cardiovascular outcomes to better characterize the emerging role of targeted cardiometabolic therapy in this distinct HFpEF phenotype.

## Review

Materials and methods

Study Design

This systematic review was conducted to evaluate the emerging role of incretin-based therapies in obesity-related heart failure with preserved ejection fraction (HFpEF), with particular emphasis on their clinical, inflammatory, structural, and cardiometabolic effects beyond glycemic control alone. The review was designed and reported in accordance with the Preferred Reporting Items for Systematic Reviews and Meta-Analyses (PRISMA) 2020 guidelines [9]. The research question was formulated using the Population, Intervention, Comparison, Outcomes (PICO) framework. The population included adult patients with obesity-related HFpEF, with or without type 2 diabetes mellitus. The intervention of interest consisted of incretin-based therapies, specifically semaglutide and tirzepatide. Comparator groups included placebo or standard background therapy. The primary outcomes included changes in heart failure-related symptoms, exercise capacity, quality of life, inflammatory markers, cardiac remodeling parameters, and cardiovascular or worsening heart failure outcomes.

Search Strategy

A comprehensive literature search was conducted across PubMed/MEDLINE, Scopus, and Web of Science to identify relevant studies published between January 2020 and April 2026. The search strategy was designed to capture contemporary randomized clinical evidence evaluating incretin-based therapies in obesity-related heart failure with preserved ejection fraction (HFpEF). Database filters were applied to restrict results to randomized controlled trials, clinical trials, pooled randomized analyses, and mechanistic substudies derived from randomized placebo-controlled trials. Search terms included combinations of Medical Subject Headings (MeSH) and free-text keywords such as “HFpEF,” “heart failure with preserved ejection fraction,” “obesity-related HFpEF,” “semaglutide,” “tirzepatide,” “GLP-1 receptor agonist,” “incretin therapy,” “cardiometabolic heart failure,” and “obesity.” Boolean operators, including “AND” and “OR,” were used to refine the search strategy. An example search strategy included: (“HFpEF” OR “heart failure with preserved ejection fraction”) AND (“semaglutide” OR “tirzepatide” OR “GLP-1 receptor agonist”) AND (“obesity” OR “cardiometabolic”). Manual screening of reference lists from relevant studies was additionally performed to identify potentially eligible articles not captured through database searching.

Eligibility Criteria

Studies were included if they were randomized controlled trials, clinical trials, prespecified pooled analyses, or mechanistic substudies derived from randomized placebo-controlled trials evaluating semaglutide or tirzepatide in adult patients with obesity-related HFpEF. Eligible studies were required to report clinically relevant outcomes related to symptom burden, exercise capacity, quality of life, inflammatory biomarkers, congestion-related physiology, structural cardiac remodeling, or cardiovascular outcomes. Both diabetic and non-diabetic obesity-related HFpEF populations were considered eligible to reflect the broad cardiometabolic spectrum of the disease phenotype. Only studies published in English within the predefined five-year period were included to ensure contemporary clinical relevance and alignment with the rapidly evolving therapeutic landscape of incretin-based therapies in obesity-related HFpEF.

Studies were excluded if they consisted of narrative reviews, editorials, conference abstracts, observational studies without interventional components, animal or preclinical investigations, or studies unrelated to obesity-related HFpEF. Trials focusing exclusively on heart failure with reduced ejection fraction, isolated obesity management without HFpEF characterization, or non-incretin pharmacologic interventions were also excluded. Secondary analyses lacking clinically or mechanistically meaningful endpoints relevant to obesity-related HFpEF pathophysiology were not included within the primary evidence synthesis.

Study Selection and Data Extraction

All retrieved records were screened independently based on titles and abstracts, followed by full-text assessment of potentially eligible articles. Duplicate studies were removed before screening. Relevant data extracted from the included studies comprised study design, patient population, intervention characteristics, comparator groups, primary phenotype focus, major clinical findings, and mechanistic or structural outcomes. Particular emphasis was placed on multidomain effects involving symptoms, exercise capacity, inflammation, congestion biology, adiposity-related remodeling, and cardiovascular-kidney interactions in order to support a phenotype-oriented synthesis of obesity-related HFpEF.

Risk of Bias Assessment

Risk of bias was assessed using the Cochrane Risk of Bias 2 (RoB 2) tool [10] for randomized controlled trials. Domains evaluated included the randomization process, deviations from intended interventions, missing outcome data, measurement of outcomes, and selective reporting. The landmark parent randomized trials were generally judged to have low overall risk of bias, whereas mechanistic secondary analyses and imaging substudies were considered to have some concerns because of their exploratory nature, smaller substudy populations, and potential selective reporting of mechanistic endpoints.

Results

Study Selection Process

**Figure 1:**
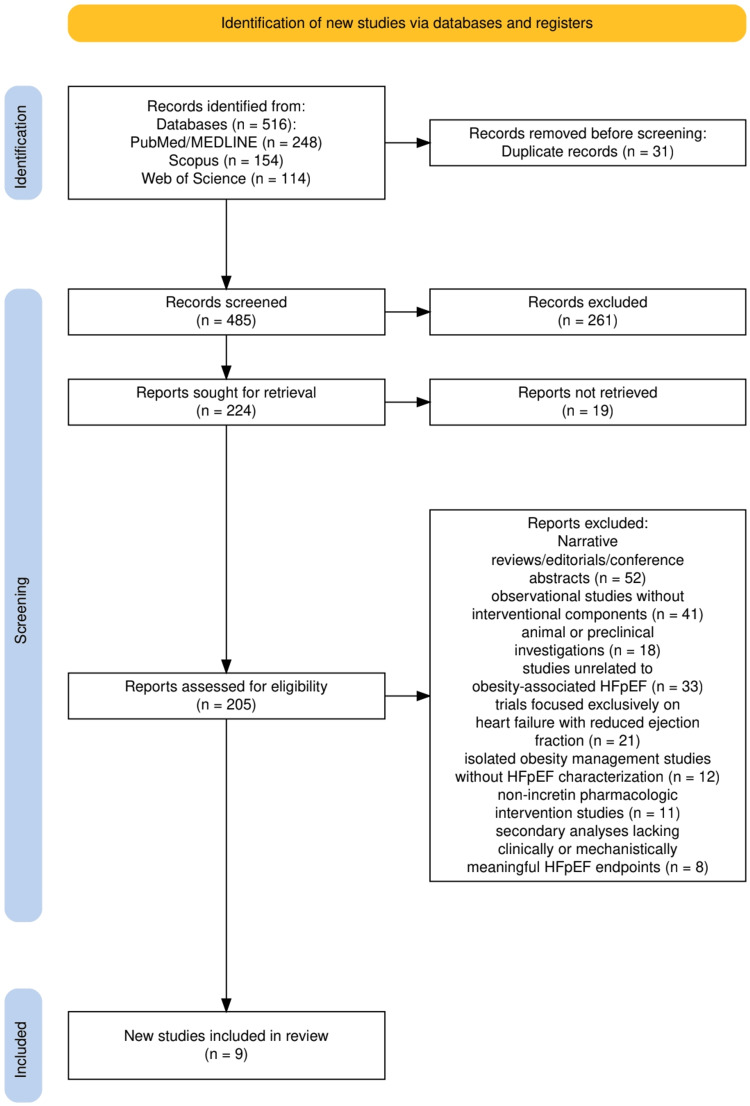
PRISMA flow diagram illustrating the study selection process for the systematic review of incretin-based therapies in obesity-related heart failure with preserved ejection fraction. PRISMA: Preferred Reporting Items for Systematic Reviews and Meta-Analyses; HFpEF: heart failure with preserved ejection fraction.

The study selection process is summarized in Figure 1. A total of 516 records were initially identified through database searching, including PubMed/MEDLINE, Scopus, and Web of Science. Following the removal of duplicate records, 485 studies underwent title and abstract screening, of which 261 were excluded. Full-text assessment was performed for 205 studies after excluding reports that could not be retrieved. Studies were excluded primarily because they consisted of narrative reviews, observational non-interventional studies, non-HFpEF populations, non-incretin therapies, or lacked clinically meaningful mechanistic endpoints relevant to obesity-related HFpEF. Ultimately, nine studies met the predefined eligibility criteria and were included in the final qualitative synthesis.

Characteristics of the Selected Studies

The studies included in the final qualitative synthesis consisted predominantly of randomized double-blind placebo-controlled trials and mechanistic substudies evaluating semaglutide or tirzepatide in patients with obesity-related HFpEF, with or without coexisting type 2 diabetes mellitus. The selected studies collectively assessed multidomain outcomes including symptom burden, exercise capacity, quality of life, inflammatory biomarkers, structural cardiac remodeling, congestion-related physiology, and cardiovascular outcomes. Several studies additionally incorporated advanced imaging modalities and biomarker analyses to explore mechanistic effects involving inflammation, adiposity-related remodeling, hemodynamic congestion, and cardiorenal interactions, thereby providing a comprehensive phenotype-oriented evaluation of incretin-based therapies in obesity-related HFpEF (Table 1).

**Table 1:** Characteristics, clinical outcomes, and mechanistic findings of included studies evaluating incretin-based therapies in obesity-related heart failure with preserved ejection fraction. HFpEF: heart failure with preserved ejection fraction; KCCQ-CSS: Kansas City Cardiomyopathy Questionnaire Clinical Summary Score; 6MWD: 6-minute walk distance; CRP: C-reactive protein; T2DM: type 2 diabetes mellitus; NYHA: New York Heart Association; CMR: cardiac magnetic resonance; LV: left ventricular; BP: blood pressure; eGFR: estimated glomerular filtration rate; STEP-HFpEF: semaglutide treatment effect in people with obesity and HFpEF; STEP-HFpEF DM: semaglutide treatment effect in people with obesity, HFpEF, and type 2 diabetes mellitus; SUMMIT: a study of tirzepatide in participants with heart failure with preserved ejection fraction and obesity.

Study	Design / Population	Intervention	Key HFpEF Phenotype Focus	Major Findings	Mechanistic / Clinical Significance
Kosiborod et al. [11]	Randomized double-blind placebo-controlled trial; 529 patients with obesity-related HFpEF	Semaglutide vs placebo	Symptomatic and functional obesity-related HFpEF phenotype	Improved KCCQ-CSS, 6MWD, hierarchical composite outcomes, CRP levels, and body weight compared with placebo	Landmark STEP-HFpEF trial establishing semaglutide as a clinically meaningful therapy in obesity-related HFpEF with benefits extending across symptoms, exercise capacity, inflammation, and metabolic burden
Kosiborod et al. [12]	Randomized double-blind placebo-controlled trial; 616 patients with obesity-related HFpEF and type 2 diabetes	Semaglutide vs placebo	Cardiometabolic obesity-HFpEF phenotype with coexisting T2DM	Improved KCCQ-CSS, 6MWD, hierarchical composite outcomes, CRP levels, and body weight compared with placebo	Demonstrates that semaglutide improves symptoms, exercise capacity, inflammatory burden, and metabolic dysfunction in obesity-related HFpEF with diabetes, supporting a multidomain disease-modifying role
Butler et al. [13]	Prespecified pooled analysis of two randomized double-blind placebo-controlled trials; 1,145 patients with obesity-related HFpEF	Semaglutide vs placebo	Multidomain obesity-related HFpEF phenotype across diabetic and non-diabetic populations	Improved KCCQ-CSS, 6MWD, hierarchical composite outcomes, body weight, and reduced CRP levels consistently across multiple clinical subgroups	Strengthens evidence that semaglutide exerts broad cardiometabolic and anti-inflammatory benefits in obesity-related HFpEF independent of major baseline demographic and clinical differences
Packer et al. [14]	International double-blind randomized placebo-controlled trial; 731 patients with obesity-related HFpEF	Tirzepatide vs placebo	Obesity-driven HFpEF clinical outcomes and health status	Reduced composite risk of cardiovascular death or worsening HF; improved KCCQ-CSS and overall health status with substantial symptomatic benefit	Landmark outcomes trial establishing tirzepatide as a potential disease-modifying therapy in obesity-related HFpEF with benefits extending beyond glycemic and weight control
Zile et al. [15]	Double-blind randomized controlled trial; 731 patients with obesity-related HFpEF	Tirzepatide vs placebo	Symptomatic burden, functional limitation, and obesity-driven HF trajectory	Reduced cardiovascular death/worsening HF risk; improved KCCQ-CSS, 6MWD, NYHA class, quality of life, patient well-being, and reduced HF medication burden	Demonstrates broad multidomain clinical improvement with tirzepatide, supporting a potential disease-modifying role in obesity-related HFpEF beyond conventional metabolic control
Verma et al. [16]	Secondary analysis of pooled randomized controlled trials; 1,145 patients with obesity-related HFpEF from STEP-HFpEF and STEP-HFpEF DM	Semaglutide vs placebo	Inflammatory obesity-HFpEF phenotype	Semaglutide improved KCCQ-CSS, 6MWD, and body weight across all CRP categories while consistently reducing CRP levels independent of baseline inflammation or magnitude of weight loss	Supports inflammation as a central mechanism in obesity-related HFpEF and suggests semaglutide may exert anti-inflammatory disease-modifying effects beyond weight reduction alone
Solomon et al. [17]	Randomized controlled echocardiography substudy; 491 patients with obesity-related HFpEF from the pooled STEP-HFpEF and STEP-HFpEF DM trials	Semaglutide vs placebo	Adverse cardiac remodeling and diastolic dysfunction in obesity-related HFpEF	Reduced left atrial remodeling, attenuated right ventricular enlargement, and improved diastolic function parameters including E-wave velocity, E/A ratio, and E/e′	Suggests semaglutide may exert disease-modifying effects through improvement in adverse remodeling and diastolic function beyond metabolic control
Kramer et al. [18]	Randomized controlled CMR substudy; 175 patients with obesity-related HFpEF from the SUMMIT trial	Tirzepatide vs placebo	Structural remodeling and adiposity-driven HFpEF phenotype	Reduced LV mass and paracardiac adipose tissue at 52 weeks; LV mass reduction correlated with weight loss and cardiac volumetric changes	Provides strong imaging-based evidence that tirzepatide may induce structural cardiac remodeling and reduce adiposity-related myocardial burden in obesity-HFpEF
Borlaug et al. [19]	Randomized controlled trial; 731 patients with obesity-related HFpEF from the SUMMIT trial	Tirzepatide vs placebo	Circulatory overload, systemic inflammation, cardiovascular-kidney end-organ injury	Reduced systolic BP, estimated blood volume, CRP, troponin T, albuminuria; improved eGFR, KCCQ-CSS, and 6MWD	Demonstrated that tirzepatide may mitigate congestion, inflammatory burden, and cardiorenal injury beyond weight reduction alone

Quality Assessment

Risk of bias assessment was performed using the Cochrane Risk of Bias 2 (RoB 2) tool for randomized controlled trials. Overall, the landmark parent clinical trials demonstrated a low risk of bias across major methodological domains, including randomization, outcome measurement, and reporting processes. Mechanistic secondary analyses and imaging-based substudies were generally assessed as having some concerns because of their exploratory nature, smaller substudy populations, and potential selective reporting of mechanistic endpoints. Nevertheless, the overall methodological quality of the included evidence remained strong and was considered appropriate for qualitative synthesis and phenotype-oriented interpretation (Table 2).

**Table 2:** Risk of bias assessment of included studies using the Cochrane Risk of Bias 2 tool. RoB 2: Risk of Bias 2 tool

Study	Suggested Tool	Randomization Process	Deviations from Intended Intervention	Missing Outcome Data	Outcome Measurement	Selective Reporting	Overall Risk of Bias
Kosiborod et al. [11]	Cochrane RoB 2	Low	Low	Low	Low	Low	Low risk
Kosiborod et al. [12]	Cochrane RoB 2	Low	Low	Low	Low	Low	Low risk
Butler et al. [13]	Cochrane RoB 2	Low	Low	Low	Low	Low	Low risk
Packer et al. [14]	Cochrane RoB 2	Low	Low	Low	Low	Low	Low risk
Zile et al. [15]	Cochrane RoB 2	Low	Low	Low	Low	Low	Low risk
Verma et al. [16]	Cochrane RoB 2	Low	Low	Low	Low	Some concerns	Some concerns
Solomon et al. [17]	Cochrane RoB 2	Low	Low	Some concerns	Low	Some concerns	Some concerns
Kramer et al. [18]	Cochrane RoB 2	Low	Low	Some concerns	Low	Some concerns	Some concerns
Borlaug et al. [19]	Cochrane RoB 2	Low	Low	Low	Low	Some concerns	Some concerns

Discussion

Opening Synthesis

Collectively, the included studies suggest that incretin-based therapies may exert broad multidomain benefits in patients with obesity-related heart failure with preserved ejection fraction (HFpEF). Across the STEP-HFpEF and a study of tirzepatide in participants with heart failure with preserved ejection fraction and obesity (SUMMIT) programs, treatment with semaglutide and tirzepatide was consistently associated with improvements in heart failure-related symptoms, physical limitations, exercise capacity, quality of life, inflammatory burden, adiposity-related parameters, and markers of adverse cardiac remodeling. Landmark investigations by Kosiborod et al. [11,12], Packer et al. [14], and Zile et al. [15] further demonstrated favorable effects on hierarchical clinical outcomes, functional status, and worsening heart failure events, while mechanistic analyses by Verma et al. [16], Solomon et al. [17], Kramer et al. [18], and Borlaug et al. [19] extended these observations to inflammatory modulation, structural remodeling, congestion biology, and cardiorenal physiology. Importantly, these effects appeared to extend beyond simple glycemic improvement or nonspecific weight reduction alone, particularly given the parallel improvements observed across inflammatory, hemodynamic, and imaging-based domains. Taken together, the current evidence raises the possibility that obesity-related HFpEF may represent a cardiometabolic and inflammation-driven phenotype that is more responsive to targeted metabolic interventions than previously appreciated.

HFpEF Heterogeneity and the Obesity Phenotype

HFpEF is increasingly recognized as a heterogeneous clinical syndrome rather than a singular pathophysiologic entity, and obesity-related HFpEF appears to represent one of its most distinct and biologically active phenotypes. Unlike traditional ischemic or hypertensive heart failure paradigms, obesity-related HFpEF is characterized by complex interactions between visceral adiposity, systemic inflammation, endothelial dysfunction, plasma volume expansion, impaired skeletal muscle energetics, concentric remodeling, and elevated filling pressures, all of which contribute to marked exercise intolerance and symptomatic limitation. The studies included in this review consistently reflected these interconnected mechanisms. Verma et al. [16] demonstrated the high prevalence and potential clinical relevance of inflammatory activation in obesity-related HFpEF, whereas Borlaug et al. [19] identified reductions in estimated blood volume, inflammatory biomarkers, and cardiorenal injury markers following tirzepatide therapy. Similarly, imaging analyses by Solomon et al. [17] and Kramer et al. [18] suggested that incretin-based therapies may favorably influence adverse remodeling, left atrial enlargement, ventricular geometry, and paracardiac adiposity. These observations are clinically important because many conventional heart failure therapies were originally developed within predominantly ischemic or reduced ejection fraction frameworks and may not adequately target the metabolic and inflammatory disturbances that appear central to obesity-related HFpEF. Consequently, the emerging success of incretin-based interventions may reflect not only symptomatic improvement, but also a more phenotype-oriented therapeutic approach to a uniquely cardiometabolic form of heart failure.

Potential Disease-Modifying Effects of Incretin-Based Therapy

Although substantial weight reduction was consistently observed across the included trials, the therapeutic effects of incretin-based interventions in obesity-related HFpEF appear to extend beyond simple caloric restriction alone. Several studies demonstrated parallel improvements across inflammatory, structural, hemodynamic, functional, and cardiorenal domains, suggesting a broader biologic impact on disease activity. In the STEP-HFpEF program, Verma et al. [16] reported significant reductions in CRP levels across varying baseline inflammatory states, whereas Solomon et al. [17] demonstrated attenuation of left atrial remodeling, improvement in diastolic indices, and favorable right ventricular structural changes following semaglutide therapy. Similarly, the SUMMIT mechanistic analyses by Kramer et al. [18] and Borlaug et al. [19] identified reductions in left ventricular mass, paracardiac adipose tissue, estimated blood volume, albuminuria, and circulating biomarkers of myocardial injury and systemic inflammation. Importantly, these changes occurred alongside improvements in exercise capacity, health status, and worsening heart failure outcomes observed in the landmark trials by Kosiborod et al. [11,12], Packer et al. [14], and Zile et al. [15].

Nevertheless, concerns have emerged regarding potential lean skeletal muscle mass reduction during substantial pharmacologic weight loss, which may hold particular relevance in HFpEF populations already predisposed to frailty, sarcopenia, and impaired exercise tolerance. Although current trials demonstrated net improvements in functional outcomes such as six-minute walk distance, the long-term implications of body composition changes and skeletal muscle preservation remain incompletely understood and warrant further investigation. The convergence of these findings across inflammatory, structural, symptomatic, and hemodynamic domains raises the possibility that incretin-based therapies may exert disease-modifying effects in obesity-related HFpEF beyond isolated glycemic improvement or nonspecific weight loss. While definitive conclusions regarding long-term remodeling reversal and mortality reduction remain premature, the current evidence increasingly supports the concept that targeted cardiometabolic interventions may favorably influence the underlying pathobiology of this HFpEF phenotype.

Inflammation as a Central Mechanism in Obesity-Related HFpEF

Chronic low-grade systemic inflammation is increasingly recognized as a central contributor to the development and progression of obesity-related HFpEF. Excess visceral and epicardial adiposity promotes adipokine dysregulation, endothelial dysfunction, oxidative stress, and activation of inflammatory signaling pathways involving interleukin-6 and CRP, all of which may contribute to myocardial fibrosis, impaired ventricular compliance, microvascular dysfunction, and progressive exercise intolerance. Within this framework, the findings reported by Verma et al. [16] are particularly important because they demonstrated not only a high prevalence of inflammatory activation in obesity-related HFpEF, but also consistent reductions in CRP levels following semaglutide therapy across varying baseline inflammatory states. Notably, mediation analyses from the STEP-HFpEF program suggested that the observed anti-inflammatory and clinical improvements were not fully explained by the magnitude of weight reduction alone, supporting the possibility that modulation of inflammatory biology may represent an additional therapeutic mechanism beyond secondary effects of adipose tissue loss. Complementary observations from Borlaug et al. [19] further support this interpretation, with tirzepatide-associated reductions in CRP occurring alongside improvements in congestion-related physiology and markers of cardiovascular-kidney injury. Collectively, these findings strengthen the emerging hypothesis that obesity-related HFpEF may represent an inflammation-mediated cardiometabolic syndrome in which incretin-based therapies exert biologically relevant effects on both systemic inflammatory pathways and downstream cardiovascular dysfunction.

Structural Remodeling and Functional Recovery

Emerging evidence from the included mechanistic substudies suggests that incretin-based therapies may influence structural and functional remodeling in obesity-related HFpEF, thereby extending their effects beyond symptomatic improvement alone. In the echocardiographic substudy of the STEP-HFpEF program, Solomon et al. [17] demonstrated attenuation of left atrial remodeling, reduction in right ventricular enlargement, and improvement in diastolic functional indices, including E-wave velocity, E/A ratio, and E/e′ following semaglutide therapy. Complementary findings from the SUMMIT cardiac magnetic resonance substudy by Kramer et al. [18] revealed significant reductions in left ventricular mass and paracardiac adipose tissue with tirzepatide treatment, suggesting favorable effects on obesity-associated myocardial remodeling and ectopic fat accumulation. These observations are particularly important because adverse atrial and ventricular remodeling are central contributors to impaired ventricular compliance, elevated filling pressures, and progressive exercise intolerance in HFpEF. Unlike many traditional heart failure therapies that primarily slow disease progression or reduce hospitalization burden, the structural improvements observed across these studies raise the possibility that incretin-based interventions may partially reverse aspects of remodeling in selected obesity-related HFpEF phenotypes. Although the long-term durability and prognostic implications of these changes remain uncertain, the consistency of imaging and functional findings across independent analyses supports the concept that targeted cardiometabolic therapy may favorably influence the underlying myocardial substrate in obesity-related HFpEF.

Congestion, Volume Expansion, and Cardiorenal Interactions

Congestion and cardiorenal dysfunction represent central yet frequently underrecognized components of obesity-related HFpEF. Excess adiposity is associated with plasma volume expansion, elevated filling pressures, sodium retention, impaired renal hemodynamics, and heightened inflammatory activation, collectively contributing to worsening exercise intolerance and recurrent heart failure exacerbations. Within this context, the mechanistic analysis by Borlaug et al. [19] provides important insight into the potential physiologic effects of tirzepatide on congestion biology and cardiovascular-kidney interactions. Tirzepatide therapy was associated with reductions in estimated blood volume, systolic blood pressure, albuminuria, CRP, and biomarkers of myocardial injury, alongside improvements in eGFR, functional capacity, and health status measures. These findings suggest that incretin-based therapies may favorably influence both hemodynamic congestion and downstream end-organ stress in obesity-related HFpEF. Importantly, the observed improvements were not confined solely to weight reduction but also involved pathways linked to inflammatory activation and cardiorenal coupling. This distinction is clinically meaningful because obesity-related HFpEF frequently behaves as a volume-expanded inflammatory syndrome in which congestion, renal dysfunction, endothelial impairment, and metabolic dysregulation coexist and perpetuate one another. Consequently, therapies capable of simultaneously addressing congestion biology and cardiometabolic dysfunction may hold particular relevance within this HFpEF phenotype.

Implications for Phenotype-Oriented HF Care

The findings of the present review have important implications for the evolving management of obesity-related HFpEF and support a broader movement toward phenotype-guided heart failure therapy. Historically, HFpEF management has relied largely on generalized symptom control and treatment extrapolated from reduced ejection fraction paradigms, despite the marked biologic heterogeneity that characterizes preserved ejection fraction syndromes. The multidomain improvements observed across the STEP-HFpEF and SUMMIT programs [11-19] suggest that obesity-related HFpEF may represent a particularly responsive cardiometabolic phenotype in which targeted metabolic interventions can influence symptoms, exercise capacity, inflammatory burden, congestion physiology, and structural remodeling simultaneously. These observations also reinforce the need for multidisciplinary models of care integrating cardiology, endocrinology, obesity medicine, nutrition, and rehabilitation strategies.

Furthermore, incretin-based therapies may eventually complement existing HFpEF therapies such as sodium-glucose cotransporter 2 (SGLT2) inhibitors and mineralocorticoid receptor antagonists, particularly in patients with prominent obesity, insulin resistance, systemic inflammation, or volume-expanded physiology. Mechanistically, the natriuretic and plasma volume-reducing effects of SGLT2 inhibitors may theoretically complement the metabolic, anti-inflammatory, and epicardial adiposity-reducing properties of GLP-1/GIP agonists, thereby supporting a more integrated multidomain therapeutic approach in obesity-related HFpEF phenotypes. Although incretin-based therapies demonstrated favorable multidomain clinical effects across the included trials, gastrointestinal adverse effects, particularly nausea, vomiting, and treatment discontinuation related to tolerability, remained among the most commonly reported safety considerations and may influence long-term adherence in clinical practice. Although current evidence does not yet justify universal adoption of incretin-based therapy across all HFpEF populations, it increasingly supports their emerging role as promising phenotype-oriented interventions in obesity-related HFpEF. Careful patient selection, longer-term outcome evaluation, and integration within broader cardiometabolic treatment frameworks will remain essential as this therapeutic field continues to evolve.

Future Directions in Obesity-Related HFpEF

The rapid emergence of incretin-based therapies in obesity-related HFpEF highlights several important directions for future investigation. First, the findings across the included studies [11-19] reinforce the need for more refined HFpEF phenotyping frameworks incorporating metabolic dysfunction, inflammatory activation, visceral adiposity, and congestion biology rather than relying predominantly on left ventricular ejection fraction alone. Such approaches may improve therapeutic stratification and identify patient subsets most likely to derive benefit from targeted cardiometabolic interventions. Second, future research should explore combination strategies integrating incretin-based therapies with SGLT2 inhibitors, mineralocorticoid receptor antagonists, structured weight reduction programs, and exercise rehabilitation, particularly given the multifactorial nature of obesity-related HFpEF. Finally, several mechanistic and prognostic questions remain unresolved. Whether incretin-based therapies can meaningfully reverse myocardial fibrosis, improve myocardial energetics, reduce arrhythmia burden, sustain long-term remodeling benefits, or influence cardiovascular mortality remains uncertain. Additional long-duration trials incorporating advanced imaging, biomarker profiling, and mechanistic cardiovascular phenotyping will therefore be important in determining whether these therapies primarily provide symptomatic improvement or truly alter the natural history of obesity-related HFpEF [20].

## Conclusions

The emerging body of evidence suggests that obesity-related heart failure with preserved ejection fraction (HFpEF) may represent a distinct cardiometabolic and inflammation-driven heart failure phenotype that responds favorably to incretin-based therapy across multiple clinical and mechanistic domains. Beyond improvements in body weight and glycemic parameters, semaglutide and tirzepatide demonstrated consistent benefits involving symptom burden, exercise capacity, quality of life, inflammatory activity, structural remodeling, congestion physiology, and cardiorenal interactions. Collectively, these multidomain effects support the evolving concept that targeted metabolic interventions may influence the underlying pathobiology of obesity-related HFpEF rather than merely providing symptomatic relief. Although important questions regarding long-term remodeling reversal, mortality reduction, and optimal integration with existing HF therapies remain unresolved, the current evidence increasingly positions incretin-based therapies as promising phenotype-oriented strategies within the broader landscape of contemporary HFpEF management.
